# Real-Time Detection Sensor for Unmanned Aerial Vehicle Using an Improved YOLOv8s Algorithm

**DOI:** 10.3390/s25196246

**Published:** 2025-10-09

**Authors:** Fuhao Lu, Chao Zeng, Hangkun Shi, Yanghui Xu, Song Fu

**Affiliations:** 1 School of Electronic Information and Electrical Engineering, Chengdu University, Chengdu 610106, China; lufuhao@cdu.edu.cn (F.L.); 212025085406020@cdu.edu.cn (H.S.); 2 Chengdu Jinjiang Electronic System Engineering Company, Ltd., Chengdu 610051, China; jecdz@jec784.com (Y.X.); 13980588836@163.com (S.F.)

**Keywords:** black-flying drones, LSTM, YOLOv8s, Adam, Monte Carlo, embedded system

## Abstract

**Highlights:**

**What are the main findings?**

- The integration of LSTM with YOLOv8s considerably improves drone detection accuracy, tracking stability, and small-object recognition in complex low-altitude environments.
- The proposed YOLOv8s-LSTM model demonstrates faster convergence and higher robustness than baseline models, as validated by numerous UAV datasets and extensive Monte Carlo sampling.
- Compared with the PC platform, the RK3588-A-embedded platform exhibits reduced computational complexity, improved positioning accuracy, and high FPS, while consuming only one-sixth of the power consumed by a PC and shrinking the model size. These features suggest significant benefits in terms of real-time processing, energy efficiency, and cost effectiveness in edge computing scenarios such as low-altitude UAV detection.

**What is the implication of the main finding?**

- This approach, which enhances real-time drone tracking capabilities, is excellent for applications requiring high precision and reliability under dynamic and challenging conditions.
- The improved adaptability of the model to varied lighting, occlusion, and scale changes suggests strong potential for deployment in practical counter-drone and low-altitude surveillance systems.

**Abstract:**

This study advances the unmanned aerial vehicle (UAV) localization technology within the framework of a low-altitude economy, with particular emphasis on the accurate and real-time identification and tracking of unauthorized (“black-flying”) drones. Conventional YOLOv8s-based target detection algorithms often suffer from missed detections due to their reliance on single-frame features. To address this limitation, this paper proposes an improved detection algorithm that integrates a long-short-term memory (LSTM) network into the YOLOv8s framework. By incorporating time-series modeling, the LSTM module enables the retention of historical features and dynamic prediction of UAV trajectories. The loss function combines bounding box regression loss with binary cross-entropy and is optimized using the Adam algorithm to enhance training convergence. The training data distribution is validated through Monte Carlo random sampling, which improves the model’s generalization to complex scenes. Simulation results demonstrate that the proposed method significantly enhances UAV detection performance. In addition, when deployed on the RK3588-based embedded system, the method achieves a low false negative rate and exhibits robust detection capabilities, indicating strong potential for practical applications in airspace management and counter-UAV operations.

## 1. Introduction

In the field of computer vision, object detection—a fundamental yet challenging undertaking [1]—has undergone considerable advancements and found extensive applications across various domains, including surveillance, autonomous driving, and industrial production [2]. Since the advent of region-based convolutional neural networks (R-CNNs) [3] that pioneered region-based deep learning for object detection, the field has evolved through the following two paradigms: two-stage detectors (e.g., Faster R-CNN [4]) emphasizing accuracy, and one-stage detectors (e.g., You Only Look Once (YOLO) [5], single-shot detectors) prioritizing real-time performance. This paradigm shift reflects the dual demand for precision and efficiency in domains such as unmanned aerial vehicle (UAV) detection, wherein both attributes are critical.

Among these applications, detecting and tracking unmanned aerial vehicles (UAVs) has garnered considerable attention owing to the growing use of drones in civilian and military contexts [6,7]. However, practical UAV detection scenarios introduce unique challenges, including scale variability—drones appear as tiny pixels at long distances but occupy large regions in close-range footage [8]; dynamic occlusion—cluttered backgrounds (e.g., urban landscapes, foliage) or inter-drone overlap degrade feature distinctiveness [9]; and need for environmental robustness—low-light, motion blurring, or adverse weather (e.g., rain and fog) impair detection reliability [10]. These challenges necessitate algorithms that balance spatial feature fidelity and temporal context awareness; existing methods have not effectively addressed this gap.

Object detection algorithms based on deep learning, particularly those in the YOLO series, have become dominant in real-time detection tasks [2]. YOLOv8 has demonstrated excellent performance in both speed and accuracy, leading to the development of variants for specific applications. YOLO-drone [11] optimized the network for detecting tiny UAV targets by introducing a high-resolution detection branch, pruning large target layers, reducing the parameters by 59.9%, and enhancing the detection speed. ITD-YOLOv8 [12] enhanced the perception of the neck network by integrating the global context and multiscale features, while replacing the standard convolution with AXConv to adaptively reduce complexity. However, these improvements focus on spatial feature extraction, thereby overlooking crucial temporal information for video-based UAV detection.

In video-related object detection, challenges such as motion blur and defocus can considerably degrade the algorithm performance. To address these issues, the blur-aid feature aggregation network (BFAN) proposed in [13] aggregates blurred features with minimal additional computation. However, it fails to capture the dynamic motion patterns of UAVs over time. Regarding UAV-captured images, existing methods often exhibit low detection accuracy and inefficient utilization of pre-trained models. The high-quality object detection method in [14] addresses these issues by employing an improved DINO framework combined with masked image modeling. It utilizes a hybrid backbone that integrates convolutional neural networks (CNNs) and vision transformers to extract global and local features more effectively. However, this method faces challenges in satisfying real-time requirements for UAV tracking.

Detecting tiny objects—common in UAV applications—has attracted considerable attention. Approaches such as Temporal-YOLOv8 [15] enhance performance by leveraging the temporal context in videos and specialized data augmentation, thereby increasing the mean average precision (mAP) from 0.465 to 0.839. DroneNet [6] introduced a feature information enhancement module to better retain object information, which can be seamlessly integrated into the backbone network. Reference [16] investigated the effectiveness of anchor-matching strategies and imbalance in anchor-based object detection, particularly for small objects. SDS-YOLO [17] improved the localization accuracy by replacing the complete intersection-over-union loss (CIoU loss) with a shape-IoU loss, thereby providing an accurate measurement of overlap between the predicted and actual bounding boxes, which is crucial for detecting UAVs with irregular shapes or in overlapping scenarios. The Adam optimizer, as demonstrated in [18] for high-speed object detection and steering angle prediction in autonomous driving control, is effective in accelerating model convergence by adaptively adjusting learning rates based on parameter gradients. Regarding integrating recurrent neural networks (RNNs), long short-term memory (LSTM)-based methods have demonstrated strong potential in processing sequential data. The correlation-aware LSTM-based modified YOLO algorithm in [19] achieved 99.7% precision in night-vision object tracking, outperforming the standard YOLOv7. The LSTM-CNN tracker in [20] maintained 92.1% ID consistency during occlusions by modeling tracking as a 17-dimensional Markov decision process state representation.

The low-light object tracking system using the correlation-aware, modified LSTM-based YOLO algorithm suggested in offers enhanced precision in object recognition and characterization under low-light conditions. This method utilizes the sequential data processing of the LSTM and combines it with the object detection of the YOLO algorithm to address object detection and tracking in low-light environments. However, similarly to other methods, it may struggle with complex dynamic scenarios such as multiple occlusions or fast-moving targets. In military and surveillance applications, the need for detecting and classifying UAV objects, such as birds, planes, and drones, is rapidly increasing [21]. However, accurately classifying distant objects that appear as small points in images remains challenging. There is an inherent tradeoff between detection accuracy and confidence, thereby making it crucial to achieve precise detection with high confidence for practical UAV detection systems.

In related studies, target detection utilizes LSTM [22]. Analogous to traditional target detection algorithms, these methods often fail to fully exploit spatial information, resulting in low detection accuracy under non-ideal conditions, such as occlusion, deformation, wear, and poor lighting. This limitation highlights the need for more advanced algorithms capable of managing complex scenarios—a challenge that is also relevant to UAV detection, wherein dynamic environments and occlusions are prevalent.

Other studies have investigated different aspects of object detection. Reference [23] explores joint salient object detection and presence prediction, highlighting the assumption in existing models that at least one salient object is present in the input image—a premise that often leads to poor saliency maps when processing pure background images. In [24], a system-integrated YOLOv8 with MOT algorithms, BoT-SORT and ByteTrack, was used to extract multitarget information, thereby improving the redetection capability and robustness to occlusions in dynamic environments. Reference [7] highlighted the importance of artificial intelligence in enhancing real-time drone detection for anti-drone systems.

Numerous studies [25,26,27,28,29,30,31,32,33,34,35] have improved the YOLOv8 framework. Reference [25] evaluated optimized YOLOv8 variants for multiscale object detection in terms of the computational cost, energy consumption, and mAP-50. Reference [26] proposed EDGS-YOLOv8, an improved lightweight UAV detection model that incorporates ghost convolution for size reduction and enhances heads for better multiscale detection. Reference [27] used the RepVGG downsampling modules in the Drone-YOLO backbone to enhance multiscale feature learning. Reference [28] introduced YOLO-GCOF, which combines dynamic group convolution with shuffle transformers for small drone feature extraction. Reference [29] presented DMFF-YOLO, which integrates components to improve small target detection for UAV aerial photography. Reference [30] developed a reparameterization feature redundancy extraction network for UAV detection and improved feature processing during downsampling. Reference [31] proposed RPS-YOLO with a recursive feature pyramid to enhance small-object detection in UAV scenarios. Reference [32] optimized YOLO for UAV detection and classification via RF spectrogram images. Reference [33] investigated fine-grained feature-based object detection using the C2f module. Reference [34] improved the small-object detection in the UAV images using YOLOv8n featuring a new loss function. In [35], HSP-YOLOv8 was proposed to address the UAV detection challenges using a small prediction head and specific convolution.

The approaches proposed in [36,37] significantly enhance detection accuracy and efficiency by integrating YOLOv8 with the RK3588 platform, offering a promising direction for UAV target detection.

With the rapid growth of the low-altitude economy, the increasing frequency of unauthorized (“rogue”) drone activities presents serious threats to public safety, airspace regulation, and flight operations. This highlights the urgent need for a drone detection and countermeasure system that offers high precision, strong robustness, and real-time performance. In response to this demand, this study proposes a UAV detection algorithm that integrates LSTM networks with YOLOv8s and is implemented on the RK3588-A embedded platform. The goal is to enhance tracking performance for dynamically moving aerial targets. The proposed method combines the robust spatial feature extraction capabilities of YOLOv8s with the temporal sequence modeling strength of LSTM, significantly improving feature representation in temporally continuous scenarios. During training, a Monte Carlo sampling strategy is employed to increase data diversity and improve the model’s generalization ability. The loss function incorporates a combination of bounding box regression loss, binary cross-entropy, and mean squared error, while the Adam optimizer is used to accelerate convergence. Simulation results confirm that the method achieves strong performance in obstacle detection accuracy, tracking stability, and error control when deployed on the RK3588-A platform. These features make it particularly well-suited for efficient detection and reduction in rogue UAVs in complex low-altitude environments, demonstrating high practical utility and deployment potential, especially in edge computing scenarios supported by the RK3588-A platform.

Advantages and limitations of the existing object tracking techniques.
**Technology Type****Advantages****Disadvantages**Blur-aid feature aggregation network (BFAN)High-precision aggregation of blurred features with high computational efficiencyLack of dynamic and multi-scale processing, limited to single application scenarioHigh-quality object detection method based on improved dino and masked image modelingExtracting features using a hybrid network for high detection accuracyComplex model, high training cost, weak real-time performanceIntegrated YOLO Algorithm with RK3588 Embedded SystemBoosts tiny-target detection, cuts parameters and speeds up.0Hurts mid-large target performance, cannot handle temporal information.YOLOv8s combined with LSTMSpatial-temporal integration boosts robust detection and tracking, with efficient training and strong generalizationComplex structure with difficult parameter tuning, relatively high computational resource requirements

The novel methods in this study—integrating YOLOv8s with LSTM, introducing an innovative loss function design, applying the ADAM optimizer, utilizing Monte Carlo randomization and RK3588-A system—address the key limitations in existing technologies across the five following areas:

(1) Enhanced Temporal Feature Capture

The original model of YOLOv8 had limited temporal feature extraction capabilities. Integrating LSTM enables dynamic temporal attention, thereby capturing UAV state variations during positioning and tracking, including addressing conventional detector limitations.

(2) Optimized Loss Function

A task-specific loss function combining boundary regression, binary cross-entropy, and mean squared error accurately reflects prediction errors, guides the precise learning of target positions and categories, and improves the detection accuracy over conventional losses.

(3) Improved Optimal Performance

The Adam optimizer, with momentum and adaptive learning rates, adjusts parameter updates, prevents gradient problems, promotes faster and more stable convergence, and reduces training time and resources compared to other optimizers.

(4) Verification of Randomization using Monte Carlo Simulations

Monte Carlo sampling provides statistical validation (mean, standard deviation, and confidence intervals) of model stability and reliability under varied conditions, thereby surpassing single-result evaluations and addressing uncertainty and generalization gaps.

(5) Embedded Hardware Co-optimization

Hardware-aware optimization of the detection algorithm is achieved through the co-design of the RK3588-A embedded platform deployment and the algorithm. The platform’s dedicated neural processing unit (NPU) accelerates efficient computation of the lightweight model and reduces algorithmic complexity, all while maintaining high detection accuracy. This hardware-algorithm co-optimization strategy addresses the performance limitations of conventional algorithms on embedded devices and offers a lightweight, deployable solution for engineering low-altitude UAV control systems.

For comparison, the optimization strategies proposed in this study are referred to as YOLOv8s-LSTM.

## 2. Materials and Methods

### 2.1. YOLOv8s Algorithm Model

YOLOv8s reframes the object detection problem as a regression task, in which each grid cell predicts bounding boxes and their confidence scores, along with the class probabilities [25].

For each bounding box, five values must be predicted: center coordinates (*x*,*y*), width and height (*w*,*h*), and confidence score. The center coordinates (*x*,*y*) represent offsets relative to the grid cell, while the width and height (*w*,*h*) are expressed as ratios relative to the entire image. The detection equation is expressed as Equation (1) as follows:

(1) $$\left\{\begin{array}{c} x=\sigma \left(t_{x}+c_{x}\right)\\ y=\sigma \left(t_{y}+c_{y}\right)\\ w=p_{w}\cdot e^{t_{w}}\\ h=p_{h}\cdot e^{t_{h}} \end{array}\right.,$$

where $\left(c_{x},c_{y}\right)$ represents the coordinates of the top-left corner of the current grid cell, $\left(p_{w},p_{h}\right)$ the dimensions of the anchor (prior) box, and $t_{x},t_{y},t_{w},t_{h}$ the offsets predicted by the network. The sigmoid function σ restricts these offsets to the [0, 1] range.

### 2.2. Confidence Prediction and Class Prediction

The confidence score indicates the probability that an object exists within the predicted bounding box and degree of overlap between the predicted bounding box and ground truth. It is generally expressed as a scalar ranging from 0–1, wherein values closer to 1 indicate a higher likelihood that the bounding box contains an object and that its localization is accurate.

The confidence score *Confidence* is expressed by Equation (2) as follows:

(2) $$Confidence=p_{r}\left(object\right)\times {IoU}_{pred}^{truth},$$

where $p_{r}\left(object\right)\;$ denotes the probability that an object is in the bounding box, while IoU denotes a greater degree of alignment between the predicted and actual object locations.

The calculation formula for IoU is expressed by Equation (3) as follows:

(3) $$IoU=\frac{\left|B_{pred}\cap B_{gt}\right|}{B_{pred}\cup B_{gt}},$$

where $B_{pred}$ denotes the predicted bounding box and $B_{gt}$ the ground truth bounding box.

Each grid cell outputs a set of class scores for its predicted bounding box, with each score corresponding to a specific object category. The class score ${Score}_{{class}_{i}}$ for a specific bounding box is computed as the product of the confidence score and class probability, as expressed in Equation (4):

(4) $${Score}_{{class}_{i}}=p_{r}\left({class}_{i}|object\right),$$

where $p_{r}\left({class}_{i}|object\right)$ denotes the probability that the object in the bounding box belongs to class *i*, given that an object is present.

To convert these scores into probabilities, assuming that there are N classes and the network outputs a raw class score vector $z=\left[z_{1},z_{2},\ldots ,z_{N}\right]$, the conversion formula is expressed by Equation (5) as follows:

(5) $$P\left(c_{i}|B_{pred}\right)=\frac{e^{z_{i}}}{\sum_{j=1}^{N}e^{z_{j}}}i=1,2,\ldots ,N,$$

where $P\left(c_{i}|B_{pred}\right)$ denotes the probability that the object within the predicted bounding box $B_{pred}$ belongs to class *I*, $z_{i}$ the raw score output by the network for class *I*, and $z_{j}$ the raw score for class *j*.

**Figure 1:**
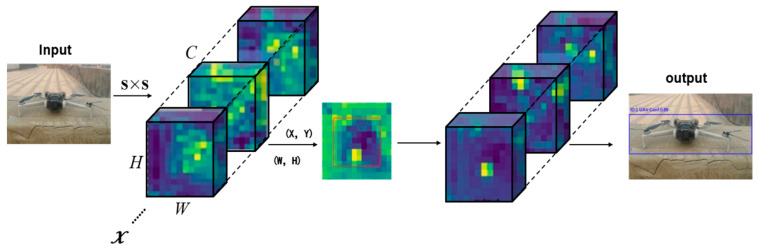
Feature map processing of YOLOv8s.

For real-time detection—an objective of this study, the confidence score and class-prediction results were used. The class prediction results for a detection box are considered only when the confidence score exceeds a certain threshold. The final detection results comprised the bounding boxes with high confidence scores and highest class probabilities. Figure 1. Feature map processing of YOLOv8s shows the feature map processing pipeline of YOLOv8s.

When using YOLOv8s for frame-by-frame drone tracking, each frame is treated as an independent image, with YOLOv8s detecting the position of the drone in every frame. However, relying on this detection can result in issues such as minor inconsistencies in detecting the same drone across different frames, including reduced accuracy owing to factors such as occlusion or changes in lighting.

**Figure 2:**
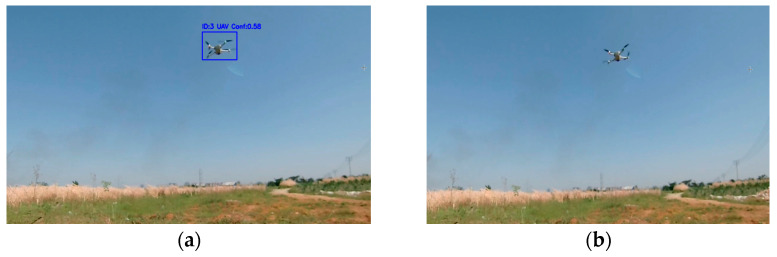
Drone detection using the YOLOv8s algorithm: (**a**) Drone detection and positioning; (**b**) missed detection frame.

In target tracking, if a frame is lost, the information for that frame is also lost, making it impossible to accurately account for the motion of the target during that interval. This can result in considerable discrepancies between the predicted and actual target positions, thereby affecting subsequent detection and association. Since real-time drone detection relies on continuous tracking, even if a target is detected in the next frame, the previously established tracking trajectory may be interrupted owing to the missing frame. As shown in Figure 2, the YOLOv8s algorithm is combined with a binocular camera for UAV detection, highlighting frame loss. Figure 2a shows that the YOLOv8s algorithm successfully identifies the drone but with a low confidence, while Figure 2b shows that the drone is not detected, thereby indicating frame loss. To address this problem, this study proposes a YOLOv8s-LSTM target-tracking model that enhances drone detection performance and mitigates tracking failure caused by video frame loss.

### 2.3. YOLOv8s-LSTM Algorithm

The optimized YOLOv8-LSTM model proposed in this paper provides an innovative approach to addressing the aforementioned issues. First, YOLOv8s is used to perform single-frame target detection on each frame of the video, accurately locating the positions of drones (and interfering targets) and outputting the coordinates of the detection boxes. Subsequently, the coordinates from consecutive frames are concatenated into a time series in chronological order and in-put into the LSTM model.

**Figure 3:**
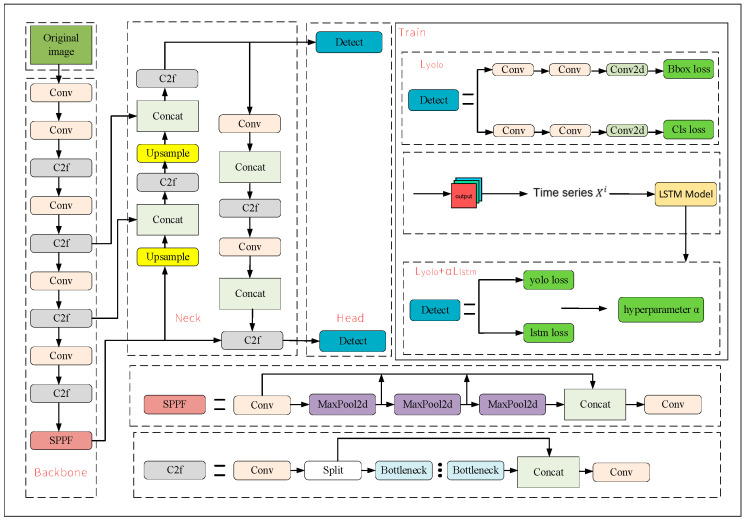
YOLOv8s-LSTM object detection architecture diagram.

Its architecture is illustrated in Figure 3. Compared with the original YOLOv8 [9], the core innovations focus on three aspects to break through the performance bottleneck of single-frame detection in complex scenarios:

(1) Enhancement of backbone and neck networks: On the basis of inheriting the efficient Cross Stage Partial (C2f) module and multi-scale feature fusion strategy of YOLOv8, this framework introduces a hierarchical progressive feature refinement mechanism with the “Convolution (Conv) + C2f” module group as the core. Through multiple rounds of feature transmission and refinement, the spatial feature fidelity of tiny UAV targets is improved.

(2) Temporal modeling module: The proposed architecture embeds an LSTM-based time-series processing branch. After the detection head of YOLOv8 outputs the bounding box coordinates of consecutive frames, these coordinates are aggregated into a time series and input into the LSTM model. This enables the model to learn the target motion patterns and overcomes the defect that YOLOv8 cannot model inter-frame dependencies.

(3) Optimization of training processes: In the training phase, the “Train” sub-module adopts a strategy of dual-path parallel “Conv + C2f” streams to enhance feature diversity, a design not available in the single-branch training of YOLOv8.

Thus, by combining the single-frame detection accuracy of YOLOv8s with the temporal sequence modeling capability of LSTM, efficient tracking of drones in complex scenarios is achieved, it can effectively reduce the occurrence of target loss.

A sequence of consecutive video frames $\left\{I_{1},I_{2,},\ldots {,I}_{t}\right\}$, where *T* denotes the total number of frames in the video or real-time surveillance footage, was processed using YOLOv8s. Each frame $I_{t}$ (*t* = 1, 2, …, *T*) is input into the YOLOv8s model for detection.

After processing frame $I_{t}$, YOLOv8s generates numerous detection boxes. For each detected object, the detection box information is represented by the vector $b_{t}^{i}=\left[x_{t1}^{i},y_{t1}^{i},x_{t2}^{i},y_{t2}^{i},c_{t}^{i},p_{t1}^{i},p_{t2}^{i},\ldots ,p_{tC}^{i}\right]$.

$\left(x_{t1}^{i},y_{t1}^{i}\right)$ and ${(x}_{t2}^{i},y_{t2}^{i})$ represent the coordinates of the top-left and bottom-right corners of the detection box, respectively; $c_{t}^{i}$ is the confidence score indicating the presence of an object within the detection box; and $p_{tj}^{i}\left(j=1,2,\ldots ,C\right)$ is the probability that the object in the detection box belongs to class *j*, where *C* denotes the total number of object classes.

To utilize this information for UAV localization using LSTM, we focused on the coordinate data from the detection boxes. Herein, partial coordinates are extracted as $X_{t}^{i}=\left[x_{t1}^{i},y_{t1}^{i},x_{t2}^{i},y_{t2}^{i}\right]$, where *i* denotes the ith UAV target. The ground truth coordinates for each UAV target are denoted as $g_{t}^{i}=\left[g_{t1}^{i},g_{t2}^{i},g_{t3}^{i},g_{t4}^{i}\right]$.

For a specific target, the coordinate information from consecutive frames is organized into a time series $X^{i}=\left[X_{t-n+1}^{i},X_{t-n+2}^{i},\ldots ,X_{t}^{i}\right]$, where $X^{i}\epsilon R^{n\times 4}\;\mathrm{and}\;X_{t}^{i}\epsilon R^{1\times 4}$ serve as the input to the LSTM [20].

When processing time-series data, the LSTM unit maintains the cell state $C_{t}$ and hidden state $h_{t}$. At each time step t, the inputs to the LSTM include the present input $x_{t}$, hidden state from the last time step $h_{t-1}$, and prior cell state $C_{t-1}$.

The LSTM algorithm centers on its cell state and three gating mechanisms: the input, forget, and output gates. These mechanisms enable LSTM to effectively learn long-term dependencies while mitigating the vanishing or exploding gradient problems associated with conventional RNNs.

The cell state is a key component of the LSTM that determines the data storage, as expressed in Equation (6):

(6) $$c_{t}=f_{t}⨀c_{t-1}+i_{t}⨀{\tilde{c}}_{t},$$

where ⨀ represents element-wise multiplication; $c_{t}$ denotes the updated cell state at the current time step, integrating the outputs of the forget gate ($f_{t}$) and input gate ($i_{t}$); ${\tilde{c}}_{t}$ is the candidate cell state generated at the current time step; and $c_{t-1}$ is the cell state from the previous time step.

The input gate determines the new information stored in the cell state, as expressed in Equation (7):

(7) $$\left\{\begin{array}{c} i_{t}=\sigma \left(W_{i}\cdot \left[h_{t-1},X_{t}^{i}\right]+b_{i}\right)\\ {\tilde{c}}_{t}=\tanh\left(W_{c}\cdot \left[h_{t-1},X_{t}^{i}\right]+b_{c}\right) \end{array}\right.,$$

where $i_{t}$ represents the output of the input gate; ${\tilde{c}}_{t}$ the candidate cell state; $W_{i}$ and $W_{c}$ the weight matrices for the input gate and candidate cell state, respectively; $b_{i}$ and $b_{c}$ the corresponding bias terms; and tanh the hyperbolic tangent function with an output range of −1 to 1.

The forget gate determines the information to be discarded from the cell state, as expressed in Equation (8):

(8) $$f_{t}=\sigma \left(W_{f}\cdot \left[h_{t-1},X_{t}^{i}\right]+b_{f}\right),$$

where $f_{t}$ represents the output of the forget gate; $W_{f}$ the weight matrix of the forget gate; $b_{f}$ the bias term of the forget gate; σ the sigmoid function with an output range of 0 to 1; $h_{t-1}$ the hidden state from the previous time step; and $x_{t}$ the input at the current time step.

The output gate determines which parts of the cell state are passed as outputs to the next time step, as expressed in Equation (9):

(9) $$\left\{\begin{array}{c} i_{t}=o_{t}=\sigma \left(W_{o}\cdot \left[h_{t-1},xX_{t}^{i}\right]+b_{o}\right)\\ h_{t}=o_{t}⨀\tanh\left(c_{t}\right) \end{array}\right.,$$

where $W_{o}$ represents the weight matrix of the output gate and $b_{o}$ the bias term of the output gate. $o_{t}$ determines how much information from the current cell state $c_{t}$ is output to the hidden state $h_{t}$.

Equation (10) expresses how the hidden state $h_{t}$, obtained from the LSTM at the current time step, is used to predict the bounding box information of the target in the next frame.

(10) $${\overset{ˇ}{x}}_{t+1}=W_{our}h_{t}+b_{out},$$

where ${\overset{ˇ}{x}}_{t+1}$ is the predicted bounding box coordinates of the target in the next frame $w_{out}$ represents the weight matrix of the fully connected layer and $b_{out}$ the bias vector.

**Figure 4:**
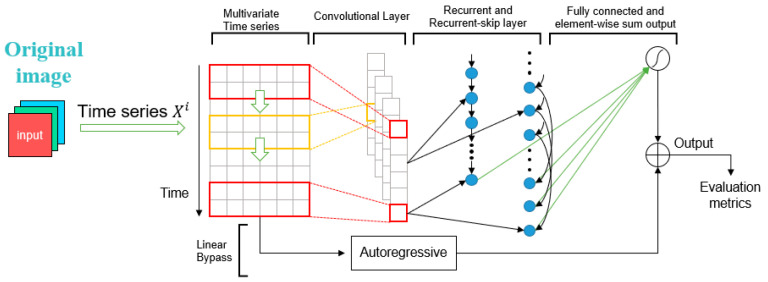
YOLOv8s-LSTM diagram.

Figure 4 shows the enhanced YOLOv8s-LSTM architecture for multivariate time-series prediction, which improves the forecasting performance by integrating convolutional layers, recurrent units, and an autoregressive component.

### 2.4. Loss Function Model

The loss function is vital in the integrated YOLOv8s and LSTM models as it gauges the difference between model predictions and ground truth labels, thereby guiding parameter updates during training [38]. As expressed in Equation (11), the combined loss function L of the model comprises two components: the detection loss $L_{yolo}$ from YOLOv8s and prediction loss $L_{lstm}$ from LSTM, scaled by a hyperparameter α to control their contributions.

(11) $$L=L_{yolo}+\alpha L_{lstm}.$$

The YOLOv8s detection loss, as expressed in Equation (12), comprises two components: the bounding-box regression loss $L_{box}$ and confidence loss $L_{conf}$:

(12) $$L_{yolo}=L_{box}+L_{conf}.$$

The bounding box regression loss employs complete intersection over union (CIoU), an enhanced IoU metric that takes into account not only the overlap between predicted and ground truth boxes, but also integrates center point distance and aspect ratio. CIoU is expressed by Equation (13) as follows:

(13) $$L_{box}=\frac{1}{N}\underset{i=1}{\overset{N}{\sum }}L_{box}^{i}=\frac{1}{N}\underset{i=1}{\overset{N}{\sum }}1-CIoU\left(b_{gt}^{i},\hat{b^{i}}\right),$$

where $b_{gt}^{i}$ denotes the ground-truth bounding box; $\hat{b^{i}}$ the predicted bounding box; and N the number of bounding boxes involved in the loss computation within a batch.

The equation aggregates the individual regression losses $L_{box}^{i}$ computed for each bounding box, which is later divided by the total number of bounding boxes N to obtain the average bounding box regression loss $L_{box}$. This guides the adjustment of the bounding box prediction parameters during model training.

The confidence loss is computed using the binary cross-entropy loss function, which measures the difference between the predictions and ground truth labels of the model. The confidence loss is expressed by Equation (14) as follows:

(14) $$L_{conf}=\frac{1}{N}\sum_{i=1}^{N}L_{conf}^{i}=\frac{1}{N}\sum_{i=1}^{N}{-y}_{c}^{i}\log\left(c^{i}\right)-\left(1{-y}_{c}^{i}\right)\log\left(1-c^{i}\right),$$

where N denotes the number of samples; $y_{c}^{i}$ the ground-truth label indicating whether an object is present in the detection box; and $c^{i}$ the predicted confidence score.

This equation computes the overall confidence loss by averaging the confidence loss across all detection boxes to optimize the confidence prediction of the detection boxes during model training.

As expressed in Equation (15), the prediction loss of LSTM is calculated using the mean squared error loss, which measures the difference between the predicted bounding box information and ground truth. The LSTM model parameters were optimized by minimizing the loss function as follows:

(15) $$L_{lstm}=\frac{1}{M}\underset{t=1}{\overset{M}{\sum }}{\left\|{\hat{x}}_{t+1}-x_{t+1}\right\|}_{2}^{2},$$

where M denotes the number of time series samples used to train the LSTM; ${\hat{x}}_{t+1}$ the predicted bounding box; $x_{t+1}$ the ground truth bounding box; and $\|.{\|}_{2}^{2}$ the squared $L_{2}$-norm that measures the distance between ${\hat{x}}_{t+1}$ and $x_{t+1}$, quantifying the magnitude of their difference.

**Figure 5:**
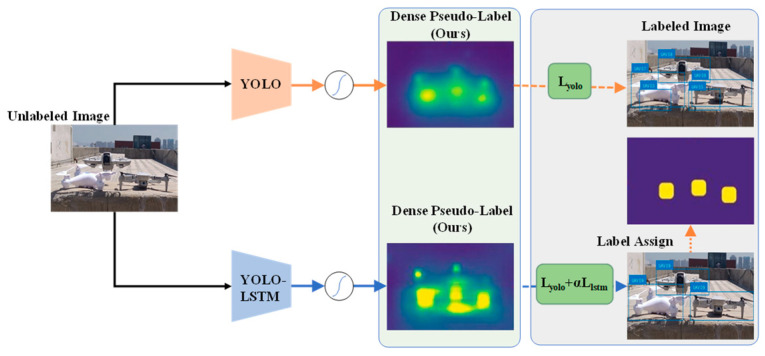
Schematic of the dense pseudo-label generation and annotation process of YOLOv8s and YOLOv8s-LSTM.

The formula computes the overall loss by averaging the distances between the predicted and ground truth values at each time step. Figure 5 illustrates the workflow of the integrated YOLO-LSTM object-detection framework, which combines the YOLO detector with a temporal LSTM model and employs a pseudo-labeling mechanism to enhance supervision and object detection.

### 2.5. Adam Optimizer

The Adam optimizer merges the benefits of momentum and adaptive learning rates [24]. It computes individualized learning rates for different parameters, enabling rapid updates and fast convergence during the initial training phases, while providing stable and controlled adjustments later to prevent overshooting due to overly large learning rates. In the YOLOv8s-LSTM framework, accurately learning spatial features via YOLOv8s and temporal sequence features via LSTM is critical. The adaptively optimized step size and direction allow the model to efficiently converge toward an optimal parameter set within a short training period.

The first-order moment estimation is defined in Equation (16) as follows:

(16) $$m_{t}=\beta_{1}m_{t-1}+\left(1-\beta_{1}\right)g_{t},$$

where $m_{t}$ denotes the first-order moment estimate; $\beta_{1}$ the decay rate for the first-order moment, generally close to 1; $m_{t-1}$ the first-order moment estimate from the previous iteration; and $g_{t}$ the gradient of the loss function with respect to parameter θ.

The second-order moment estimation is expressed by Equation (17) as follows:

(17) $$v_{t}=\beta_{2}v_{t-1}+\left(1-\beta_{2}\right)g_{t}^{2},$$

where $v_{t}$ denotes the second-order moment estimate; $\beta_{2}$ the second-order moment decay rate; $v_{t-1}$ the second-order moment estimate prior to iteration; and $g_{t}^{2}$ the squared gradient of the loss function L with respect to parameter θ.

Because the initial values of $m_{t}$ and second-order moment estimate $v_{t}$ are generally set to zero vectors, they are biased toward zero in the early iterations. Hence, bias correction is necessary.

The bias-corrected first-order moment estimate is expressed in Equation (18) as follows:

(18) $$\hat{m_{t}}=\frac{m_{t}}{1-\beta_{1}^{t}}.$$

The bias-corrected second-order moment estimate is expressed using Equation (19) as follows:

(19) $$\hat{v_{t}}=\frac{v_{t}}{1-\beta_{1}^{t}},$$

where *t* denotes the iteration step. As the number of iterations increases, the effect of the bias correction gradually decreases.

Equation (20) expresses the update of the model parameters $\theta_{t}$ using the bias-corrected first-order and second-order moment estimates as follows:

(20) $$\theta_{t}=\theta_{t-1}-\eta \frac{\hat{m_{t}}}{\sqrt{\hat{v_{t}}}+\epsilon },$$

where η denotes the learning rate and ϵ a constant.

Using these steps, the Adam optimizer iteratively updates the model parameter θ using the gradient of the loss function, thereby minimizing the total loss function $L=L_{yolo}+\alpha L_{lstm}$ and optimizing the YOLOv8s-LSTM model.

Integrating LSTM with YOLOv8s offers considerable advantages for object detection and localization. An analysis of the object parameter distributions shows that the model adapts well to position and scale variations, effectively managing diverse scenarios. Evaluations using key metrics—mAP50, mAP50-95, precision, and recall—demonstrated considerable improvements in the detection accuracy and object capture capability. Loss analysis further confirmed that the model converged faster and was more stable during training. The Monte Carlo sampling results verified that the model maintains stable performance and high accuracy under different conditions.

## 3. Experimental Results

### 3.1. Experimental Settings

In this study, the compiled dataset served as the primary benchmark for experimental validation. In computer vision, selecting an appropriate dataset is crucial, as it directly affects the robustness and generalization of the model. To satisfy the growing demand for UAV detection and tracking, numerous specialized UAV datasets have been developed. Below is an overview of some representative and widely used UAV datasets.

(1) MAV-VID [39]: In this dataset, the UAV targets were distributed along the horizontal axis with relatively concentrated positions and noticeable displacements. The detected objects were very small, with an average size of a mere 0.66% of the total image area.

(2) Purdue [40]: This dataset comprises 50 videos captured by cameras mounted on high-speed drones, focusing on three UAV targets. Owing to its relatively uniform background and limited variation in UAV types, it is more suitable for research on small UAV target tracking rather than complex UAV detection tasks.

(3) Anti-UAV [41]: This dataset comprises 318 fully annotated videos with dual-modality information, including visible and infrared data. Although most UAV instances were concentrated near the center of the frame, they exhibited diverse motion patterns. The dataset was particularly designed to enhance the performance of vision-based detectors in low-light and nighttime environments.

**Figure 6:**
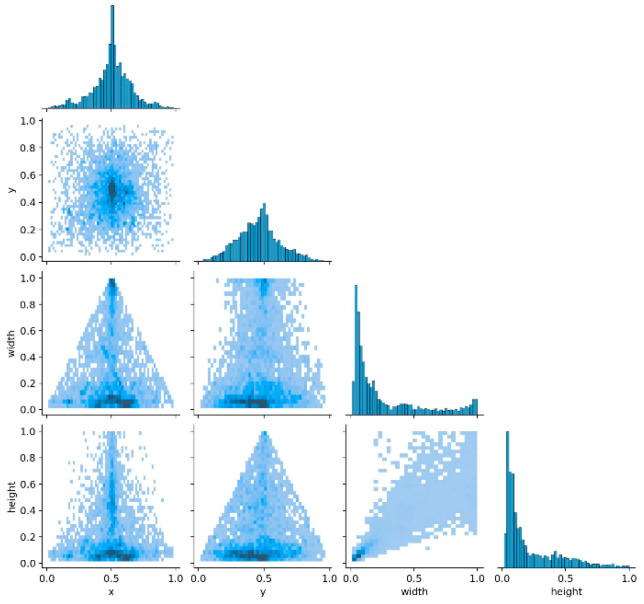
Position distribution of the dataset.

Compared to the aforementioned datasets, that used in this study features a more scattered distribution of UAV targets. As indicated by the scatter points and distribution characteristics in the figure, the targets were relatively evenly distributed in the horizontal and vertical directions. The scatter plot of the target positions referenced from the center of Figure 6 illustrates this broad and balanced spatial distribution.

During dataset construction, deliberate and systematic diversification was applied across UAV types, scene backgrounds, lighting conditions, and weather variations, rather than relying on random aggregation. This high degree of diversity not only enabled model training to cover more complex and realistic scenarios but also considerably enhanced the robustness of the model, thereby highlighting the crucial role of dataset diversity in this study.

Compared to the aforementioned datasets, the dataset in this study exhibits a more dispersed UAV target distribution. Figure 6 presents the distribution characteristics of the target positions (*x* and *y* coordinates relative to the image center) and sizes (width and height), along with quantitative statistics. For positions, the *x*-coordinate mean is 0.52 (*σ_x_* = 0.28) and the *y*-coordinate mean is 0.49 (*σ_y_* = 0.26), with the 95% confidence interval ellipse covering *x* ∈ [0.15, 0.89] and *y* ∈ [0.12, 0.86], indicating a uniform distribution in both directions with moderate dispersion and no aggregation. For sizes, the mean width is 0.31 (*σ* = 0.12) and the mean height is 0.29 (*σ* = 0.11), with reasonable variations covering diverse UAV scales.

These quantified distributions confirm the diversity of the dataset in both positions and sizes. Constructed through systematic design rather than random aggregation, it incorporates diverse UAV types, scenes, lighting conditions, and weather patterns. The above metrics demonstrate its coverage of complex, comprehensive scenarios, which is critical for enhancing model generalization and robustness.

**Figure 7:**
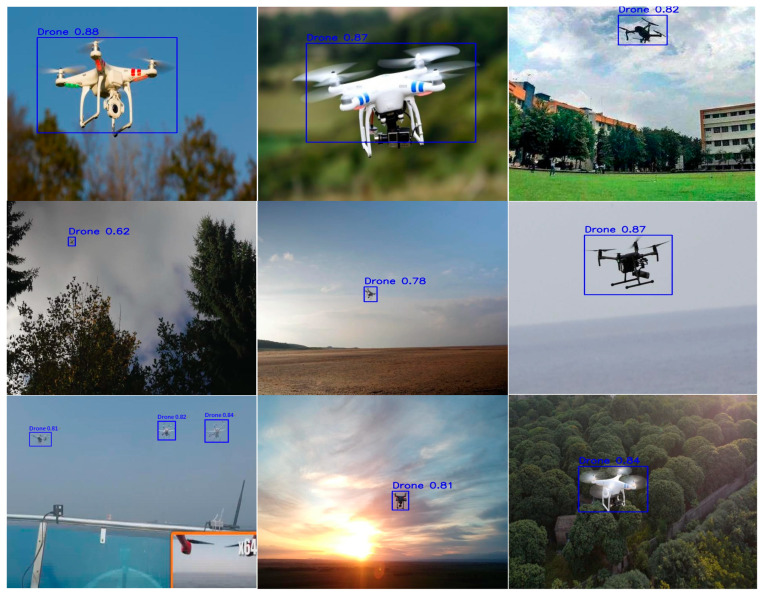
Detection images and annotations in our dataset. Objects within bounding boxes stand for the drone detection targets specified in dataset annotations.

The training dataset comprised 6670 images, the validation dataset 1906 images, and the test dataset 953 images, each with a resolution of 640 × 640 pixels. Figure 7 shows the detection images and their annotations in our dataset. Data were split into a 70:20:10 ratio for training, validating, and testing. All the models were trained on a GPU workstation for 500 epochs. Table 1 presents the hardware configuration of the personal computer used in this experiment, while Table 2 presents the software versions applied.

**Figure 8:**
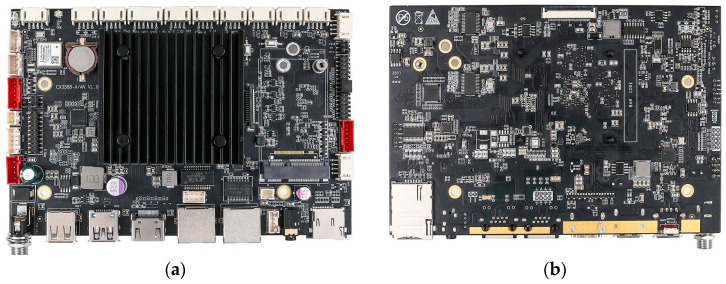
(**a**) RK3588-A front view; (**b**) RK3588-A reverse view.

In order to verify the deployment advantages of the algorithms in real engineering scenarios, RK3588-A embedded platform of RXMicro is selected for comparative analysis with a general-purpose PC platform, as shown in Figure 8. The front and back hardware layouts of the RK3588-A intuitively show its heterogeneous computing architecture characteristics. The comparison verifies the significant advantages of the proposed algorithm in terms of detection accuracy, inference speed and complexity optimization. Table 3 details the PC hardware configuration used in the experiment.

### 3.2. Tracking Performance Metrics Analysis

In this study, we selected YOLOv8s as the baseline model and conducted comparative analyses on both a PC and the RK3588-A platform. Table 4 presents a comparison of the output results of various models on the PC platform., YOLOv8m achieved an accuracy of 95.9% in this hardware environment, outperforming the baseline model YOLOv8s. However, constrained by its larger parameter count, its frame rate (in FPS) performance is relatively inferior. In contrast, our proposed YOLOv8s-LSTM model maintains higher accuracy while featuring a more compact model size and faster detection speed, thus demonstrating significant advantages in UAV target detection tasks.

Furthermore, although YOLOv5s exhibits outstanding frame rate and parameter compactness, its mean average precision (mAP) does not meet the requirements of high-precision applications. While YOLOv11 and YOLOv13 show excellent accuracy, their large parameter sizes result in low frame rates. Experimental verification indicates that the dataset used in this study attains a more balanced performance when deployed on the YOLOv8s model running on the RK3588-A platform.

**Figure 9:**
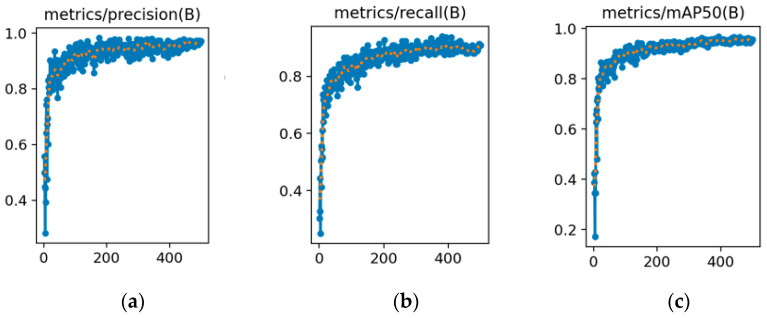
Drone detection performance of YOLOv8s: (**a**) Precision; (**b**) recall; (**c**) mAP50 metrics.

**Figure 10:**
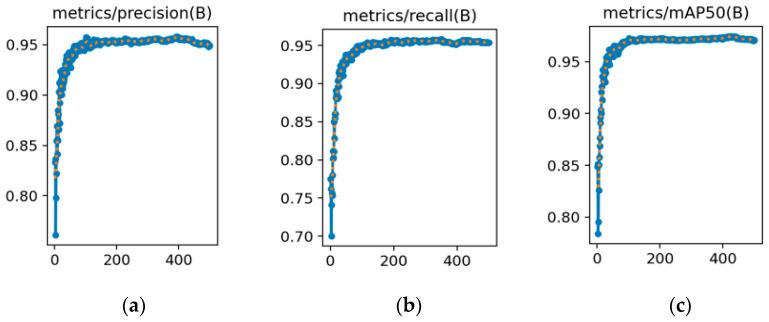
Drone detection performance of YOLOv8s-LSTM: (**a**) Precision; (**b**) Recall; (**c**) mAP50 metrics.

**Figure 11:**
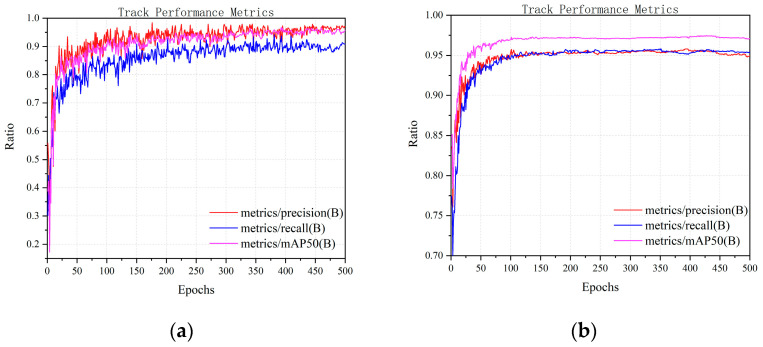
Drone detection performance metrics: (**a**) YOLOv8s; (**b**) YOLOv8s-LSTM.

Figure 9 and Figure 10 show the trends of the three key performance metrics for YOLOv8s and YOLOv8s-LSTM across training iterations, Figure 11 compares the performance of both methods in UAV target detection, including the evaluation metrics of precision, recall, and mAP50 over the training course, where the x-axis (abscissa) represents the number of training epochs, ranging from 0 to 500, and the y-axis (ordinate) denotes the parameter “ratio” within the range of [0, 1]. Table 5 shows that in the 1st training epoch, YOLOv8s-LSTM significantly outperforms the comparison models in terms of mAP50 and precision. Table 6 shows that at the 100th training epoch, YOLOv8s-LSTM outperforms the comparison models across all three metrics: mAP50, recall, and precision. Table 7 shows the number of training epochs required to reach the maximum metrics and indicates that YOLOv8s-LSTM attains the highest mAP50, recall, and precision considerably faster, achieving higher values than comparative models across all three metrics.

The YOLOv8s-LSTM model achieves higher mAP50, recall, and precision than the comparative models within a shorter training duration, demonstrating faster convergence speed and superior tracking performance.

### 3.3. Analysis of Loss Parameters

**Figure 12:**
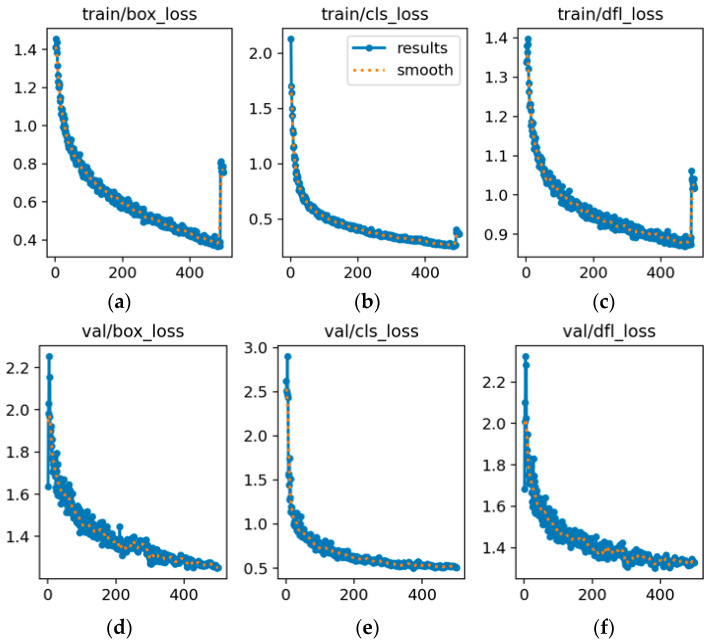
Training and validation loss for YOLOv8s: (**a**) Bounding box training loss; (**b**) classification training loss; (**c**) detection training loss; (**d**) bounding box validation loss; (**e**) classification validation loss; (**f**) detection validation loss.

**Figure 13:**
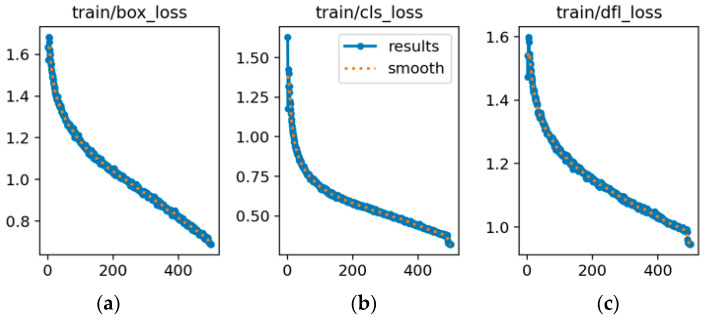

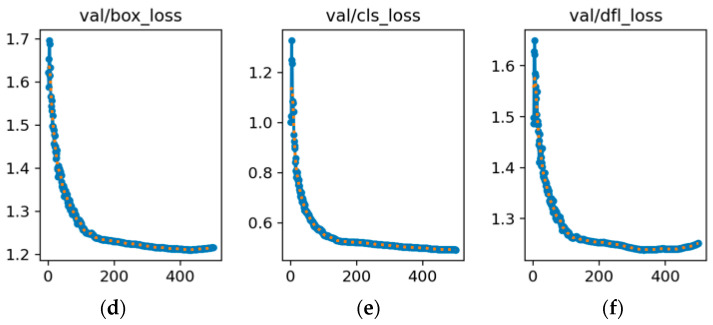
Training and validation loss for YOLOv8s-LSTM: (**a**) Bounding box training loss; (**b**) classification training loss; (**c**) detection training loss; (**d**) bounding box validation loss; (**e**) classification validation loss; (**f**) detection validation loss.

**Figure 14:**
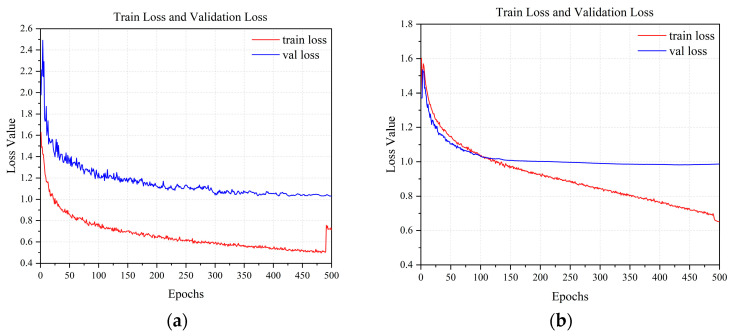
(**a**) Training and validation loss for YOLOv8s; (**b**) training and validation loss for YOLOv8s-LSTM.

As shown in Figure 12, YOLOv8s exhibits a declining training loss but a stable validation loss at approximately 1.1–1.2 between epochs 100 and 150, with a widening gap indicating potential overfitting. Figure 13 illustrates that YOLOv8s-LSTM achieves a lower and more stable validation loss alongside a smaller training-validation loss gap, suggesting better generalization and balanced performance. Figure 13 compares the training and validation losses of the two models. As shown in Figure 14a, the loss initially decreases sharply but later fluctuates, indicating instability during training. Figure 14b shows smoother, lower-loss, and less-fluctuating trends, demonstrating effective optimization and stable convergence, where the x-axis represents the number of training epochs (0–500) and the y-axis denotes the loss value.

**Figure 15:**
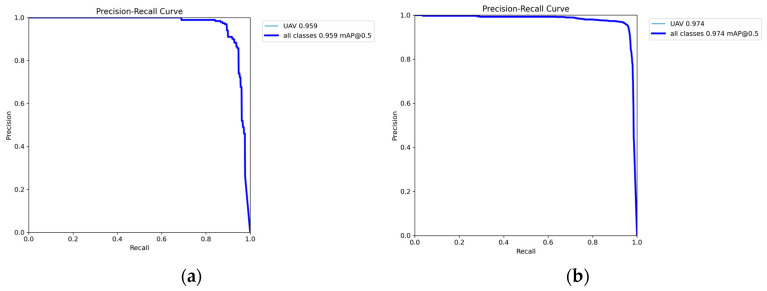
Precision–recall curve for (**a**) YOLOv8s and (**b**) YOLOv8s-LSTM.

Figure 15a shows the YOLOv8s precision-recall curve (slightly curved) with an mAP@0.5 of 0.959 for the UAVs detection, thereby indicating some false positives or missed detections. Figure 15b shows the YOLOv8s-LSTM precision–recall curve and an mAP@0.5 of 0.974, highlighting the enhanced temporal perception, accuracy, and stability. These results confirm that YOLOv8s-LSTM outperforms YOLOv8s in terms of training robustness and detection precision. To test the model’s performance across different drone flight distances, six videos were recorded, each corresponding to a distinct drone flight distance interval, for the purpose of conducting tests. Table 8 shows that the model exhibits the most stable confidence in targets within the range of 20 to 60 m, while achieving the highest confidence in targets within the 20–30 m range.

### 3.4. Experimental Validation on Det-Fly Aerial Dataset

To further evaluate the detection capability of YOLOv8s-LSTM in diverse UAV scenarios, we employed the DetFly dataset. This dataset, captured by another drone, contains over 13,000 images of flying UAV targets. Although it features only a single type of drone, it addresses the limitations of conventional datasets collected from fixed viewpoints by offering a variety of perspectives and target orientations, including upward, downward, and below-the-horizon views. These complex shooting angles pose greater challenges for object detection, particularly owing to considerable variations in the UAV attitudes. Hence, this dataset is used to assess the robustness and adaptability of the model to complex scene variations. During the experiments, we followed the data-partitioning protocol described in [44], and the detailed experimental results are presented in Table 9.

### 3.5. Performance Comparison of Small Object Detection Algorithms

We comprehensively assessed the YOLOv8-LSTM model for small-object detection across diverse scenarios using the VisDrone2019 dataset [45], a widely recognized benchmark in this field. The dataset contains numerous small-object instances spanning diverse environments and is divided into 10 categories, including pedestrians, cars, vans, and buses. As presented in Table 10 we compared the proposed YOLOv8-LSTM to the baseline YOLOv8n using key performance metrics, including precision, recall, mAP@0.5, and mAP@0.5:0.95. The results demonstrated that YOLOv8-LSTM improved mAP@0.5 and mAP@0.5:0.95 by over 5%, including considerable gains in precision and recall. These findings indicate that, compared to the original YOLOv8n, the proposed model enhances the detection accuracy considerably while effectively reducing missed detections, particularly for small objects.

### 3.6. Monte Carlo Sampling Validation

By performing 1000 Monte Carlo samples on the metric parameters of 500 training epochs, the metric values under different sample conditions were simulated, resulting in the sampling mean, standard deviation, and confidence intervals. Compared to a single raw metric value, these statistics offer a more comprehensive reflection of the metric characteristics under different sampling conditions. The sampling mean approximates the original value, thereby validating the effectiveness of the sampling process. This standard deviation measures the dispersion of the sampling results, wherein a smaller value indicates a more stable model performance. The confidence interval denotes the possible range of the true value at a given confidence level, thereby offering a basis for evaluating the reliability of the performance of the model [46,47].

For a certain tracking index denoted as I (mAP50 and precision), after N=1000 samplings, the value obtained in each sampling is $I_{i}$ (i=1,2,…,1000). The mean value $\bar{I}$ of the sampling index is expressed using Equation (21):

(21) $$\bar{I}=\frac{1}{N}\sum_{i=1}^{N}I_{i}.$$

As expressed in Equation (20), the standard deviation σ used to measure the degree of dispersion of the sampling index values is expressed as follows:

(22) $$\Sigma =\sqrt{{\left(\frac{1}{N}\sum_{i=1}^{N}I_{i}-\bar{I}\right)}^{2}}.$$

The sampling index values follow a normal distribution (in the case of large samples, this holds based on the central limit theorem). The equations for calculating the 95% confidence interval are as follows:

(23) $$\mathrm{Lower}\;\mathrm{limit}:\;\bar{I}-z_{\frac{\alpha }{2}}\frac{\sigma }{\sqrt{N}},$$

(24) $$\mathrm{Upper}\;\mathrm{limit}:\;\bar{I}+z_{\frac{\alpha }{2}}\frac{\sigma }{\sqrt{N}},$$

where α=1−0.95=0.05 and $z_{\frac{\alpha }{2}}$ denotes the quantile of the standard normal distribution. For a 95% confidence interval, $z_{\frac{\alpha }{2}}\approx 1.96$, representing the z value corresponding to the right-hand tail area of $z_{\frac{\alpha }{2}}=0.025$ in the standard normal distribution.

**Figure 16:**
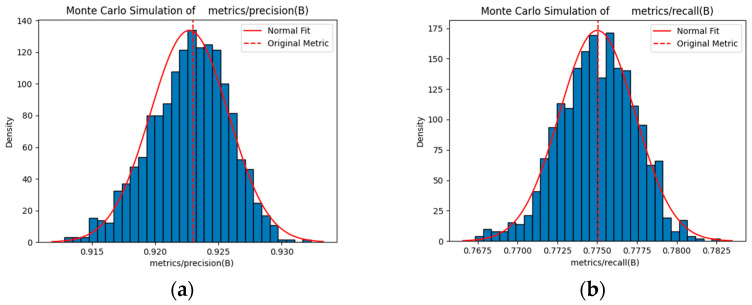

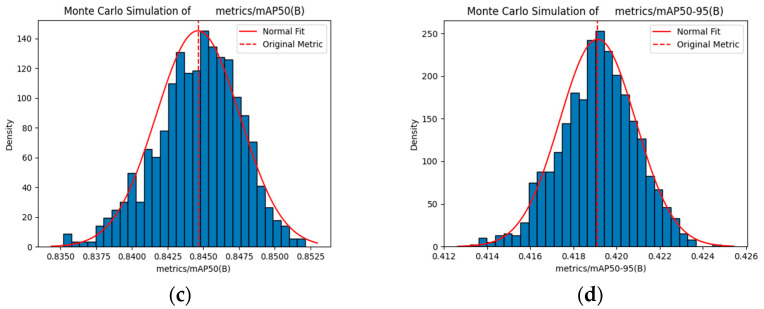
Monte Carlo simulation for YOLOv8s: (**a**) Precision, (**b**) recall, (**c**) mAP50, and (**d**) mAP50-95 metric sampling.

**Figure 17:**
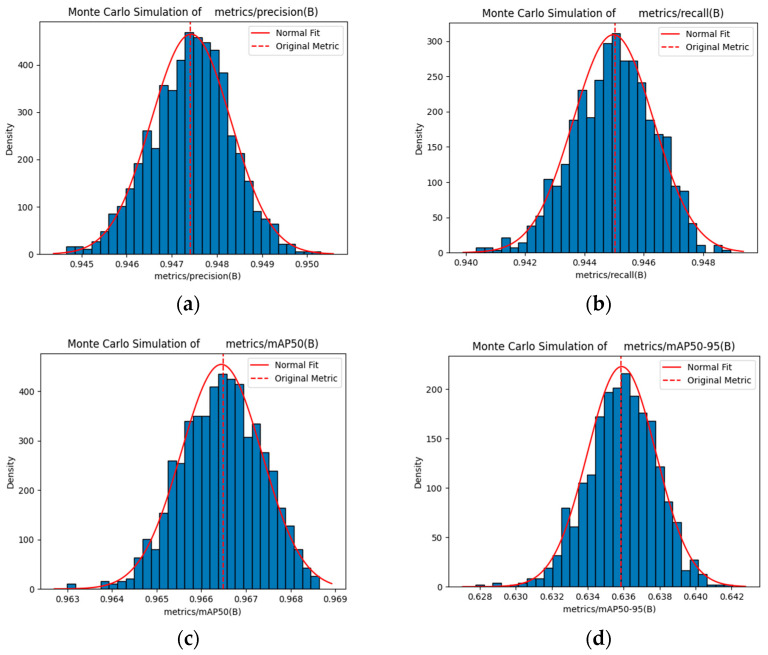
Monte Carlo simulation for YOLOv8s-LSTM: (**a**) Precision, (**b**) recall, (**c**) mAP50, and (**d**) mAP50-95 metric sampling.

Figure 16 and Figure 17 show the Monte Carlo simulation results for the performance metrics of the compared models using the standard object detection assessment metrics. Table 11 and Table 12 presents the raw and sampled mean values for mAP50, mAP50-95, precision, and recall. The close alignment between these values indicates that the 1000 Monte Carlo samples represent the true performance, thereby exhibiting minimal deviation and confirming the reliability of the sampling process.

The data comparison in the aforementioned tables shows that the YOLOv8s model exhibits considerable fluctuations in mAP50, precision, and recall across standard object detection evaluation metrics. Further, the mean values obtained after sampling using Monte Carlo simulation are minimal, indicating that the performance stability of the model varies across different detection scenarios and may underestimate its real performance. Contrarily, YOLOv8s-LSTM exhibits less fluctuations across all metrics, thereby demonstrating a more stable and consistent performance.

### 3.7. Visualization

**Figure 18:**
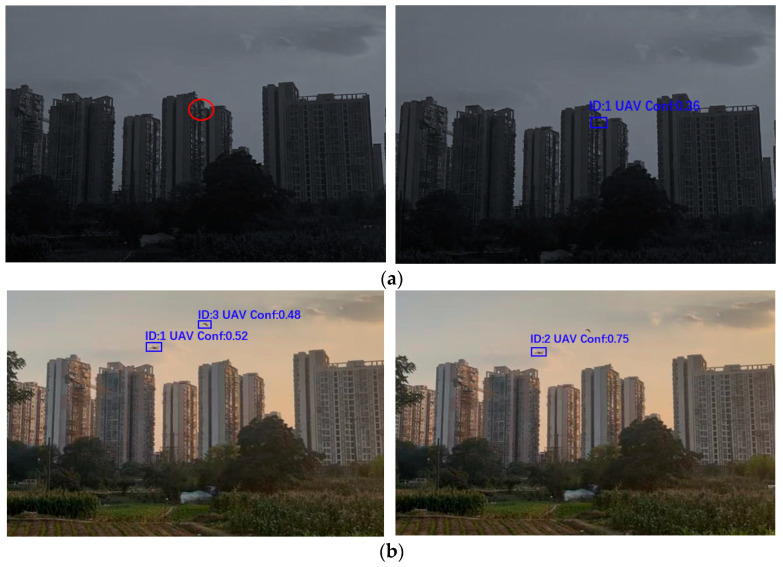
Output between the YOLOv8s model (**left**) and proposed model (**right**) on (**a**) a low-light sample image and (**b**) a normal-light sample image. The red ellipses represent missed detections. The proposed model works better under low-light and when occluded objects are detected.

Figure 18 shows the comparative output results of the model proposed in this study. These images visually compare the detection performance of the proposed method versus that of the baseline YOLOv8s model in varying environmental conditions YOLOv8s was selected for comparison owing to its number of parameters and complexity, ensuring a fair and intuitive performance comparison.

**Figure 19:**
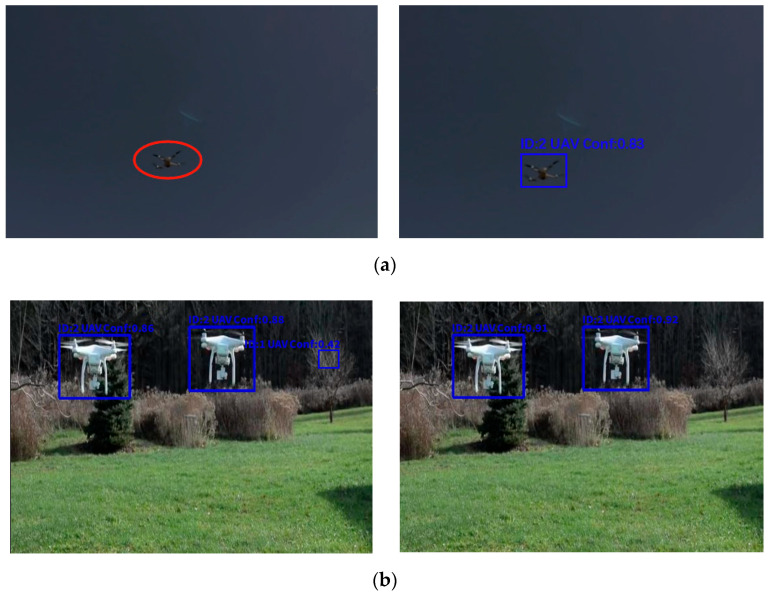
Comparison between the YOLOv8s model (**left section**) and proposed model (**right section**). Output results on the sample image in a (**a**) dark scene and (**b**) bright scene. The red-colored ellipses represent the missed objects. The proposed model demonstrates superior performance under lighting conditions.

Figure 18 and Figure 19 show the detection results of both models across the different scenarios. As shown in Figure 18, our proposed model improves the false detection rate by leveraging the small-target detection, which enhances the localization accuracy for tiny targets. Further, the LSTM structure improved the stability of the model under illumination variations and environmental disturbances, while the ADMA optimizer improved the image capture performance. Based on these improvements, our method demonstrates superior adaptability to complex natural scenes. As shown in Figure 18b, the YOLOv8s model misclassifies a bird as a drone, whereas the output from our model shows no false detection, thereby validating its robustness and accuracy in small-target recognition and complex environments. The baseline model tends to produce false detections in multi-target detection scenarios, Manually labeled images use red ellipses to mark missed detections in Figure 18a (left). Conversely, our proposed model successfully identifies the drone under these adverse conditions, demonstrating stronger target detection capabilities. To evaluate the performance of the YOLOv8s-LSTM, we manually labeled images from real-world scenarios to obtain a standard dataset for testing, which was classified according to weather conditions, as detailed in Table 13.

Figure 19 illustrates the superior performance of the proposed model’s efficacy in detecting drones under challenging scenarios like low illumination, occlusion, and long-range distances. In these scenarios, wherein the drones are small, distant, and poorly illuminated, the YOLOv8s baseline model demonstrates limited detection capability. Although both models occasionally missed detections, our model achieved a more stable detection performance owing to its higher recall rate. Figure 19a (left) shows that the baseline model misses drones under low-light and long-distance conditions, Manually labeled images use red ellipses to mark missed detections. Similarly, it fails to detect drones that are partially occluded owing to the viewing distance. In contrast, Figure 19a (right) shows our proposed model successfully identifies the drone under these adverse conditions, demonstrating stronger target detection capabilities.

### 3.8. Embedded Implementation and Visualization

**Figure 20:**
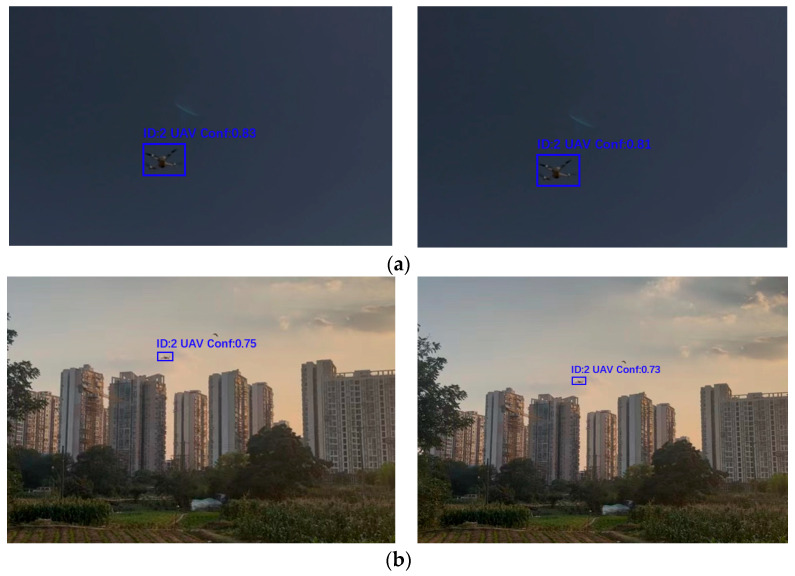
Output of the PC (**left panel**) and the RK3588-A (**right panel**) on (**a**) Output results on the sample image in a dark scene and (**b**) a sample image under normal light conditions.

In order to verify the adaptability of the model in real deployment scenarios, an improved algorithm was deployed on the general-purpose PC (Figure 20, left) and the embedded RK3588 platform (Figure 20, right), and the results of drone target detection were compared. In the output of the RK3588 platform, the target bounding box fits the drone parcel more optimally, and the confidence level is improved, the image details are presented more clearly, and the contour of the drone and the texture are more completely retained.

Table 14 presents a comparison of detection confidence between the two platforms across varying UAV flight distances. Table 15 compares the detection indexes of the general-purpose PC and the RK3588-A platform. It is evident that the RK3588-A, with NPU acceleration and model quantization, improves the FPS from 59.3 to 68.6, reduces the floating-point operation volume to 6.4G, drastically reduces the power consumption, and lowers the precision rate and the recall rate, which ensures the reliability of detection and achieves a significant improvement in the real-time performance and energy-efficiency ratio and also provides a better solution for the edge deployment of the low-altitude UAV detection.

Benefiting from the NPU acceleration and scenario adaptability of RK3588-A, the model, when deployed at the edge, effectively increases FPS, reduces computational complexity, and enhances the visualization of target localization. This provides hardware support for real-time, embedded UAV control systems in low-altitude economic scenarios.

## 4. Conclusions and Future Works

The tracking data were visualized by analyzing the distribution and correlation of the target parameters, including the position (X and Y coordinates), scale (width, height), and category. This provides an intuitive representation of the input data for the model. By optimizing the structure, modifying the loss model during training, and incorporating the Adam optimizer, the results show that integrating the LSTM with the YOLOv8s model considerably enhances adaptability to target locations and scale, enabling the system to manage a broader and more diverse range of detection scenarios.

We offer a thorough analysis of the UAV detection metrics, including key metrics such as mAP50, mAP50-95, precision, and recall. These metrics measure the detection accuracy and target capturing of the model from different perspectives. Furthermore, detailed analysis of the training and validation losses revealed that the integration of LSTM with the YOLOv8s model offers notable advantages in terms of the speed of a faster loss convergence and improved final stability, thereby highlighting the high training efficiency and stability of the model. When deployed on the RK3588-A embedded platform, the model benefits from the platform’s 6TOPS NPU acceleration, leading to reduced computational complexity and improved inference speed.

To further validate the reliability of the UAV detection metrics, 1000 sampling iterations were conducted using the Monte Carlo method. By analyzing the average, standard deviation, and 95% confidence interval of the sampling metrics, the integration of the LSTM and YOLOv8s models can maintain stable performance and provide high accuracy under different sampling conditions. Notably, on the RK3588-A platform, these metrics demonstrate consistent stability even in high-density UAV scenarios, with FPS fluctuations within 5% and positioning errors controlled within the 95% confidence interval.

The comprehensive analyses, including real-world deployment on the RK3588-A platform, demonstrate that the integration of LSTM with YOLOv8s models offers considerable advantages in terms of tracking and localization performance, with tangible improvements in real-time inference speed, computational efficiency, and positioning precision. This result fully highlights the strong application potential of the integrated model in edge computing scenarios, especially suitable for low-altitude airspace management systems built on the RK3588-A embedded platform, and also indicates that it possesses prominent practical value in airspace management and anti-UAV operations.

## Figures and Tables

**Table 1:** Hardware configuration of the PC.

Unit	Hardware Configuration
CPU	Core i7-13620H CPU @ 2.40 GHz (Max 4.9 GHz)
GPU	NVIDIA GeForce RTX 4070 Ti
Memory	DDR5—5600 RAM 16 GB × 2
Storage	Kingston SNV2S1000G 1TB SSD

**Table 2:** Software version.

Software	Version
Python	3.8.0
Pytorch	1.10.1
Anaconda	23.7.2
JetPack	1.0.0

**Table 3:** Hardware configuration of RK3588-A.

Unit	Hardware Configuration
CPU	Quad-core ARM Cortex-A76
GPU	Mail-G610 MC4
NPU	6TOPS
Memory	LPDDR5 16 GB
Camera	KS1A552-D Binocular

**Table 4:** Comparison of model performance.

Model	Model Size	mAP50	FPS (Tasks/s)
YOLOv5s [42]	14.4 MB	0.921	98.1
YOLOV8n [11]	5.96 MB	0.94	96.2
YOLOV8s [11]	21.4 MB	0.953	62.5
YOLOV8m [11]	197 MB	0.959	39.8
YOLOV11s [18]	37.8 MB	0.968	58.3
YOLOV13s [43]	25.6 MB	0.969	55.6
YOLOV8s-LSTM	18.6 MB	0.973	63.8

**Table 5:** Performance metrics of the comparative model at Epoch = 1.

Metrics (Epoch = 1)	YOLOv5s	YOLOv11	YOLOv13	YOLOv8s	YOLOv8s-LSTM
MAP50	(1, 38.6%)	(1, 40.2)	(1, 42.3)	(1, 38.8%)	(1, 84.9%)
Recall	(1, 31.1%)	(1, 84.1)	(1, 83.9)	(1, 84.2%)	(1, 77.5%)
Precision	(1, 55.3%)	(1, 57.4)	(1, 55.5)	(1, 55.8%)	(1, 83.3%)

**Table 6:** Performance metrics of the comparative model at Epoch = 100.

Metrics (Epoch = 100)	YOLOv5s	YOLOv11	YOLOv13	YOLOv8s	YOLOv8s-LSTM
MAP50	(100, 90.6%)	(100, 91.2)	(100, 90.1)	(100, 90.2%)	(100, 97.0%)
Recall	(100, 84.1%)	(100, 81.8)	(100, 82.1)	(100, 84.2%)	(100, 94.9%)
Precision	(100, 89.6%)	(100, 88.5)	(100, 88.6)	(100, 89.8%)	(100, 95.4%)

**Table 7:** Performance metrics of the comparative model under the maximum value.

Metrics (Epoch, Max)	YOLOv5s	YOLOv11	YOLOv13	YOLOv8s	YOLOv8s-LSTM
MAP50	(386, 92.6%)	(362, 95.8)	(366, 96.4)	(382, 96.8%)	(156, 97.5%)
Recall	(375, 93.1%)	(375, 93.9)	(373, 93.8)	(378, 94.1%)	(148, 95.8%)
Precision	(380, 93.3%)	(391, 95.1)	(379, 95.2)	(381, 94.8%)	(146, 95.8%)

**Table 8:** Performance of the YOLOv8s-LSTM model at varying distances.

Test Case	Expected	Confidence	Drone Detection Range
Test Video 1	Drone	0.86	<20 m
Test Video 2	Drone	0.96	20–30 m
Test Video 3	Drone	0.95	30–40 m
Test Video 4	Drone	0.86	50–60 m
Test Video 5	Drone	0.73	70–80 m
Test Video 6	Drone	0.51	>80 m

**Table 9:** Comparison Experiments on the Det-Fly Dataset.

Model	Precision	Recall	mAP50	mAP95
YOLOv8s	0.919	0.868	0.913	0.63
YOLOv8s-LSTM	0.973	0.948	0.946	0.65

**Table 10:** Comparison experiments on the VisDrone2019 dataset.

Model	Precision	Recall	mAP50	mAP95
YOLOv8s	0.413	0.308	0.289	0.165
YOLOv8s-LSTM	0.432	0.334	0.314	0.181

**Table 11:** Statistical analysis of the tracking metric sampling data on YOLOv8s.

Sampling Metrics	MAP50	MAP50-95	Precision	Recall
Original Metric Values	0.844655	0.419117	0.923007	0.775076
Mean of Sampling Metrics	0.844618	0.419129	0.922698	0.775013
Standard Deviation	0.002937	0.001763	0.000309	0.002380
95% Confidence Interval	[0.8382, 0.8498]	[0.8382, 0.8498]	[0.9160, 0.9281]	[0.7701, 0.7792]

**Table 12:** Statistical analysis of the tracking metric sampling data on YOLOv8s-LSTM.

Sampling Metrics	MAP50	MAP50-95	Precision	Recall
Original Metric Values	0.966495	0.635847	0.947408	0.945027
Mean of Sampling Metrics	0.966490	0.635807	0.947380	0.944970
Standard Deviation	0.000898	0.001822	0.000902	0.001329
95% Confidence Interval	[0.9747, 0.9783]	[0.6321, 0.6391]	[0.9455, 0.9490]	[0.9422, 0.9473]

**Table 13:** Distribution and mean average metric of label images.

Weather	Number	MAP50	Precision	Recall
sunny	500	92.1%	92.8%	92.2%
cloudy	500	91.3%	91.9%	91.2%
rainy	300	85.8%	86.3%	85.6%

**Table 14:** Performance of PC and RK3588-A at varying distances.

Test Case	PC Confidence	RK3588-A Confidence	Drone Detection Range
The video 1	0.82	0.81	<20 m
The video 2	0.92	0.90	20–30 m
The video 3	0.90	0.88	30–40 m
The video 4	0.83	0.80	50–60 m
The video 5	0.69	0.68	70–80 m
The video 6	0.44	0.42	>80 m

**Table 15:** PC and RK3588-A Performance Comparison.

Model	FPS	FLOPs	NPU	Precision	Confidence	mAP50
PC	59.3	11.3 G	/	0.892	0.77	0.887
RK3588-A	68.6	6.4 G	6TOPS	0.881	0.75	0.885

## Data Availability

The data supporting the findings of this study are available from the corresponding author upon request.
